# Binder‐Free Phosphorus‐Modified Multiphase Ni–Co Sulfide Nanoarchitectures with Sea‐Urchin Morphology for High‐Capacity Hybrid Supercapacitors and Practical Applications

**DOI:** 10.1002/cssc.202500588

**Published:** 2025-07-01

**Authors:** Ampasala Surya Kiran, Edugulla Girija Shankar, Manchi Nagaraju, Jae Su Yu

**Affiliations:** ^1^ Department of Electronics and Information Convergence Engineering Institute for Wearable Convergence Electronics Kyung Hee University 1732 Deogyeong‐aero, Gihung‐gu Yongin‐si Gyeonggi‐do 17104 Republic of Korea

**Keywords:** energy storage, hybrid supercapacitors, nickel–cobalt sulfides, phosphorization, sea‐urchin‐like morphologies

## Abstract

Owing to the high demand for advanced energy storage, the exceptional characteristics of transition metal sulfides have attracted great interest from researchers. Herein, phosphorus (P)‐encapsulated nickel–cobalt sulfides (NCSs) prepared via a hydrothermal process and phosphorized in a tube furnace to produce P@NCS_20_ (with 20 mM sulfur source) are reported. The P@NCS_20_ material is synthesized through a meticulous multistep process, ensuring precise control of its material properties. Successful incorporation of P significantly enhances the electrochemical performance of the electrode. The electrochemical evaluation demonstrates the superior performance of the P@NCS_20_ electrode. Cyclic voltammetry and galvanostatic charge–discharge curves reveal enhanced oxidation/reduction behavior and high areal capacity. Electrochemical testing shows high redox reactions and decreased electrode–electrolyte resistance, and the P@NCS_20_ electrode can last for over 10000 cycles. A hybrid supercapacitor (HSC) constructed with P@NCS_20_ and activated carbon/nickel foam electrodes exhibits a specific capacitance of 95.93 F g^−1^ at 3 mA cm^−2^. This HSC device demonstrates outstanding cycling stability, and it can efficiently store solar energy and continuously power electronic devices. This work emphasizes the potential of P@NCS_20_ electrode materials in advanced energy storage systems and provides a systematic and simple synthesis procedure with important implications for sustainable energy applications.

## Graphical abstract

A binder‐free, phosphorus‐modified Ni–Co sulfide electrode with sea‐urchin‐like architecture is synthesized via hydrothermal phosphorization. The electrode exhibits high areal capacity, enhanced redox kinetics, and superior energy density in hybrid supercapacitors. The device demonstrates real‐time energy harvesting and storage, highlighting its promise for advanced and self‐powered energy storage systems.

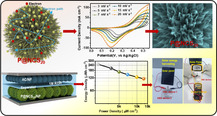

## 1 Introduction

The escalating global energy demand, depletion of fossil fuel reserves, and intensifying environmental concerns have accelerated the transition toward renewable energy sources. Technologies such as solar, wind, and nuclear power are increasingly being adopted; however, their intermittent nature necessitates the development of efficient and scalable energy storage systems to ensure a stable energy supply.^[^ ^1^ ^]^ To address this issue, electrochemical energy storage systems have been developed, and supercapacitors have emerged as an efficient energy storage technology, alongside lithium‐ion batteries, lithium–sulfur batteries, and sodium‐ion batteries, because of their high power density, extended cycle life, safety, and quick charge/discharge characteristics.^[^ ^2^ ^]^ Supercapacitors stand out for their high power density, long cycle life, operational safety, and rapid charge–discharge capabilities. Despite these advantages, their relatively low energy density (typically 5–10 Wh kg^−1^) limits their broader practical application.^[^ ^3^ ^]^ Conventional supercapacitors, particularly electrical double‐layer capacitors, store energy through electrostatic ion adsorption on electrode surfaces, offering rapid response but limited charge capacity due to surface‐constrained ion storage. In contrast, pseudocapacitors, which utilize reversible Faradaic redox reactions, offer higher energy densities, leveraging the bulk of the active material for charge storage.^[^ ^4^ ^]^

Among pseudocapacitive materials, transition metal‐based compounds, especially binary nickel–cobalt sulfides (NCSs), have emerged as promising candidates due to their high theoretical capacitance, environmental benefit, low cost, and synergistic redox behavior.^[^ ^5^, ^6^ ^]^ However, their practical deployment is often hindered by drawbacks such as poor electrical conductivity, limited electrochemical stability, and structural degradation during prolonged cycling.^[^ ^7^ ^]^ To solve these problems, engineering nanostructures with high specific surface areas is a widely adopted strategy, as it enhances electrode–electrolyte interactions and facilitates ion diffusion. Moreover, surface modification techniques, such as phosphorization, have demonstrated significant potential in improving the conductivity, electrochemical activity, and cycling performance of metal sulfides.^[^ ^7^ ^]^

Recent efforts in energy storage research have focused on developing high‐performance electrode materials that can deliver both high areal capacity and long‐term stability. For instance, a binder‐free Co_3_S_4_/nickel foam (NF) electrode achieved an areal capacity of 543.7 μAh cm^−2^ via a two‐step hydrothermal synthesis,^[^ ^8^ ^]^ while NiMn—OH nanosheets demonstrated 718 μAh cm^−2^ at 3 mA cm^−2^,^[^ ^9^ ^]^ with a relatively more complex fabrication protocol involving multiple solvothermal treatments and component integration. Similarly, hierarchical Ni_0.54_Co_0.46_O_2_ architectures, grown on biomass carbon fiber cloth, were synthesized by a hydrothermal method.^[^ ^10^ ^]^ These unique nanosheet structures showed a Faradaic areal capacity of 438 μAh cm^−2^. Performance is highly dependent on lattice defects and crystal nonstoichiometry. A battery‐type electrode using a core–shell hybrid structure was prepared on NF using NiCo_2_S_4_@polypyrrole (PPy), and these conductive PPy layers interconnected with NiCo_2_S_4_ exhibited an areal capacity of 800 μAh cm^−2^.^[^ ^11^ ^]^ The multistep coating using conductive polymer adds up synthetic and synthesizing complexity with increased costs. These conductive polymers can suffer from poor cycling stabilities due to volumetric changes and degradation over time. Although these systems show promise, they often require intricate synthesis procedures, binder additives, or multicomponent assembly, which can compromise scalability and increase cost. Although these systems show promise, they often require intricate synthesis procedures, binder additives, or multicomponent assembly, which can compromise scalability and increase cost.

In contrast, our work reports a facile, economical, and binder‐free strategy for synthesizing a phosphorus‐modified Ni–Co sulfide electrode (P@NCS_20_) material directly on NF substrate via low‐temperature hydrothermal and phosphorization steps. This method eliminates the use of polymeric binders or conductive additives, simplifying the overall process and improving electrical connectivity. The resulting P@NCS_20_ electrode exhibited an outstanding areal capacity, significantly surpassing the performance of the systems. This enhancement is attributed to the synergistic interaction of the multiphase Ni–Co–P–S matrix, optimized sea‐urchin‐like morphology, and enhanced redox‐active surface area. These combined advantages make the P@NCS_20_ electrode a strong candidate for next‐generation high‐performance supercapacitors with practical scalability and cost‐effectiveness. The characterization and application of the P@NCS_20_ electrode were conducted for high‐performance energy storage. The P@NCS_20_ electrode was fabricated via a simple hydrothermal process, followed by phosphorization, aimed at enhancing its electrochemical properties through improved conductivity and surface reactivity. The resulting electrode exhibited a distinctive sea‐urchin‐like nanostructure, which offers a large electroactive surface area and efficient ion transport pathways. Electrochemical evaluation revealed a high areal capacity and outstanding cycling stability, with high retention after 10000 consecutive galvanostatic charge–discharge (GCD) cycles. To demonstrate its practical potential, the P@NCS_20_ electrode was assembled into an hybrid supercapacitor (HSC) device using activated carbon (AC) as the negative electrode. The fabricated HSC delivered a high areal capacitance, alongside remarkable areal energy and power densities. Furthermore, the device successfully powered small‐scale electronic components, highlighting its applicability in real‐world energy storage applications.

## 2 Results and Discussion

**Figure 1:**
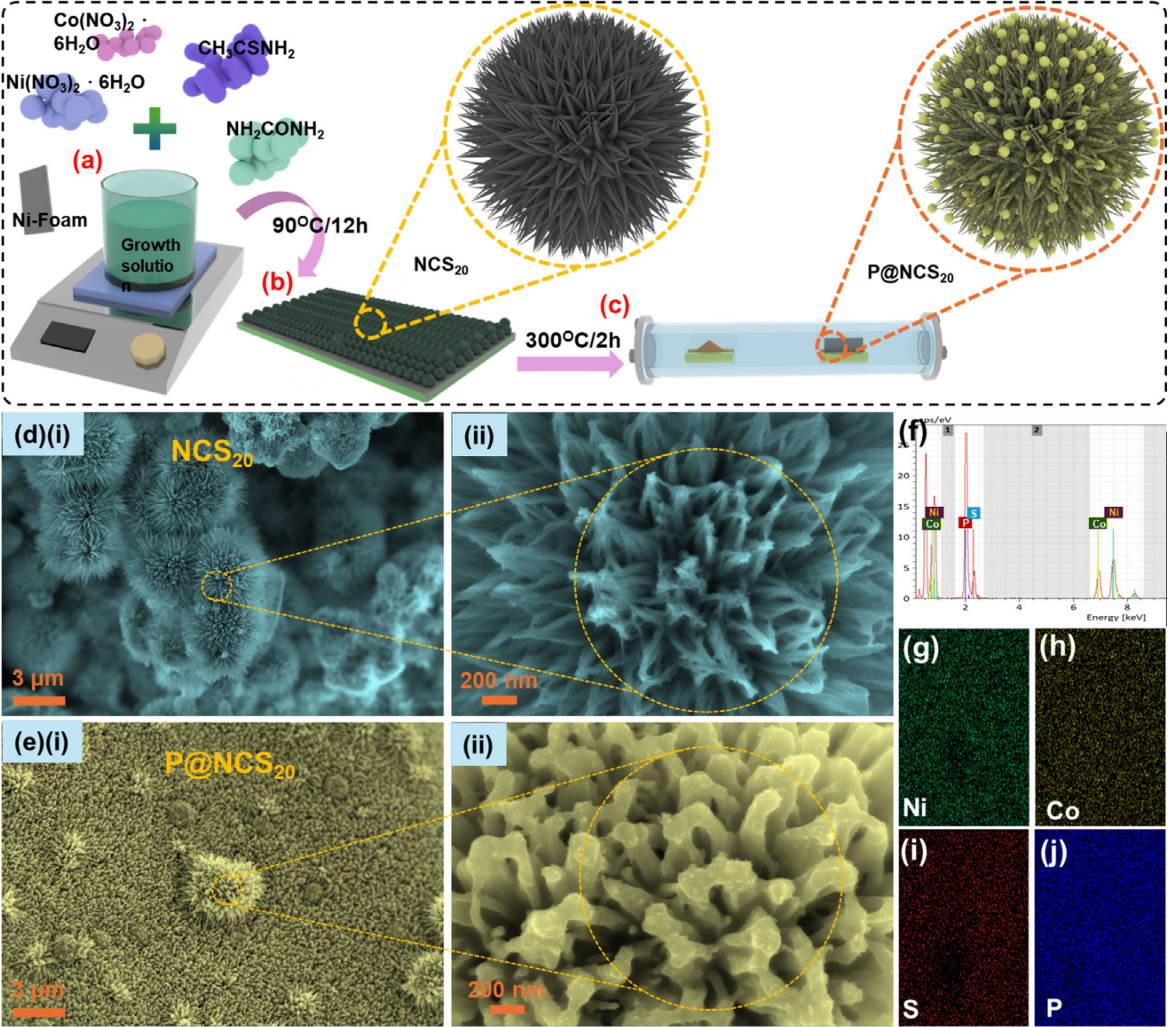
a–c) Schematic diagram of the P@NCS_20_ material in oven‐based synthesis. d), (i) Low‐ and (ii) high‐magnification FE‐SEM images of the NCS_20_ material. e), (i) low‐ and (ii) high‐magnification FE‐SEM images of the P@NCS_20_ material. f) EDS spectrum and g–j) elemental mapping images of the P@NCS_20_ material.

The P@NCS_20_ electrode material was synthesized via a meticulous multistep process, ensuring precise control of the material properties (**Figure** **1**(a)). Initially, the precursor solution was prepared by dissolving Ni, Co, and S sources in deionized water (DIW). CH_3_CSNH_2_ was the S source at the concentrations of 10, 20, and 30 mM. This solution was continuously stirred at 100 rpm to ensure a uniform dispersion of the metal ions and the S source. The cleaned NF substrate was then carefully immersed in the precursor solution to ensure full coverage. The substrate and solution were subsequently subjected to a thermal treatment in an electric oven kept at a consistent temperature of 90 °C for 12 h. This thermal process helped the formation of a uniform coating on the NF substrate. After treatment, the resultant electrode, as depicted in Figure 1(b), was rigorously washed multiple times with ethanol and DIW to eliminate any unreacted species or impurities and then dried to prepare it for the next step. Subsequently, an NCS_20_ electrode was obtained. As in low temperatures (<120 °C), the aggregation process progresses gradually, and the metallic ions (Ni, Co, and S) from the growth solutions start to interact with each other and form a sea‐urchin‐like morphology, which is a result of various factors like van der Waals forces, hydrophobic attraction, and Ostwald ripening.^[^ ^12^, ^13^ ^]^ From a kinetic perspective, the initial homogeneous precipitation conditions necessitate that each precursor particle has adequate free space to create nanosized particles. Subsequently, these nanoparticles unite and undergo Ostwald ripening processes. The growth mechanism along specific directions is then altered by the adsorption of modifiers such as water, surfactants, anions (OH^−^ and S^2−^), and cations (Ni^2+^ and Co^2+^). The objective of this process is to reduce the free energy of the system so that agglomeration occurs, and the structures come together, allowing for permeable growth.^[^ ^14^, ^15^ ^]^ These structures subsequently undergo slow growth into nanorods through dissolution–recrystallization processes. This series of processes ultimately results in the formation of urchin‐like microspheres.

Phosphorization was performed to further modify the electrode. The pre‐synthesized NCS_20_ electrode was exposed to 0.5 g of NaH_2_PO_2_·H_2_O, serving as the P source. This step was performed in a tubular furnace under a N_2_ atmosphere to prevent oxidation. The temperature was carefully maintained at 300 °C for 2 h, with a heating rate of 5 °C min^−1^, as shown in Figure 1(c). This phosphorization process was critical for incorporating P into the electrode structure, thus forming the final P@NCS_20_ electrode. The resulting P@NCS_20_ electrode material was extensively characterized to ascertain its structural and electrochemical properties. The incorporation of P was confirmed using various analytical techniques, indicating the successful modification of the electrode. The enhanced electrochemical performance observed in subsequent tests suggests that the P@NCS_20_ electrode has significant potential for applications in energy storage and conversion systems. The detailed process parameters and systematic approach to synthesis highlight the reproducibility and reliability of this fabrication method.

Figure 1(d) shows field‐emission scanning electron microscope (FE‐SEM) images of the optimized P@NCS_20_ material. The FE‐SEM images revealed an agglomerated morphology in the cluster of nanorods, as depicted in Figure 1(d)(i) and (ii). This morphology can be attributed to the introduction of S and ammonium into the solution and the alkaline nature of the growth solution, which exerts a favorable influence on electrochemical investigations. Figure 1(e)(i) and (ii) presents the low‐ and high‐magnification FE‐SEM images of the NCS_20_ material after phosphorization. The phosphorization process induces a significant transformation in the material surface morphology, altering it from stacked nanosheets to well‐defined nanorods, which collectively forms a one‐dimensional architecture. This structural evolution significantly enhances electrochemical performance by providing a more accessible pathway for the rapid migration of electrolytic ions. It offers a more accessible pathway for the rapid migration of electrolytic ions. The elongated nanorod structure not only enhances the active surface area but also reduces ion diffusion resistance, resulting in efficient charge transport and enhancing the electrode's overall energy storage capacity.^[^ ^12^ ^]^ The urchin‐shaped P@NCS_20_ electrode and NCS_20_ electrodes’ reduced charge‐transfer resistance clearly illustrate the important influence of morphology on electrochemical behavior. Charge transfer in both the P@NCS_20_ electrode and NCS_20_ electrode is improved by the urchin morphology's hierarchical structure and porosity‐enriched architecture. Furthermore, our results prove that morphological characteristics are essential for boosting electrochemical activity, even though anion exchange plays a critical role in increasing the electrode material's capacitance. Energy‐dispersive X‐ray spectroscopy (EDS) was used to ascertain the elemental distribution within the P@NCS_20_ material. The EDS spectrum confirmed the presence of various elements in the P@NCS_20_ material at different intensities, thereby confirming the existence of Ni, Mn, S, and P, as illustrated in Figure 1(f). Notably, these elements were uniformly dispersed throughout the P@NCS_20_ material, as evidenced by the elemental mapping images in Figure 1(g)–(j).

**Figure 2:**
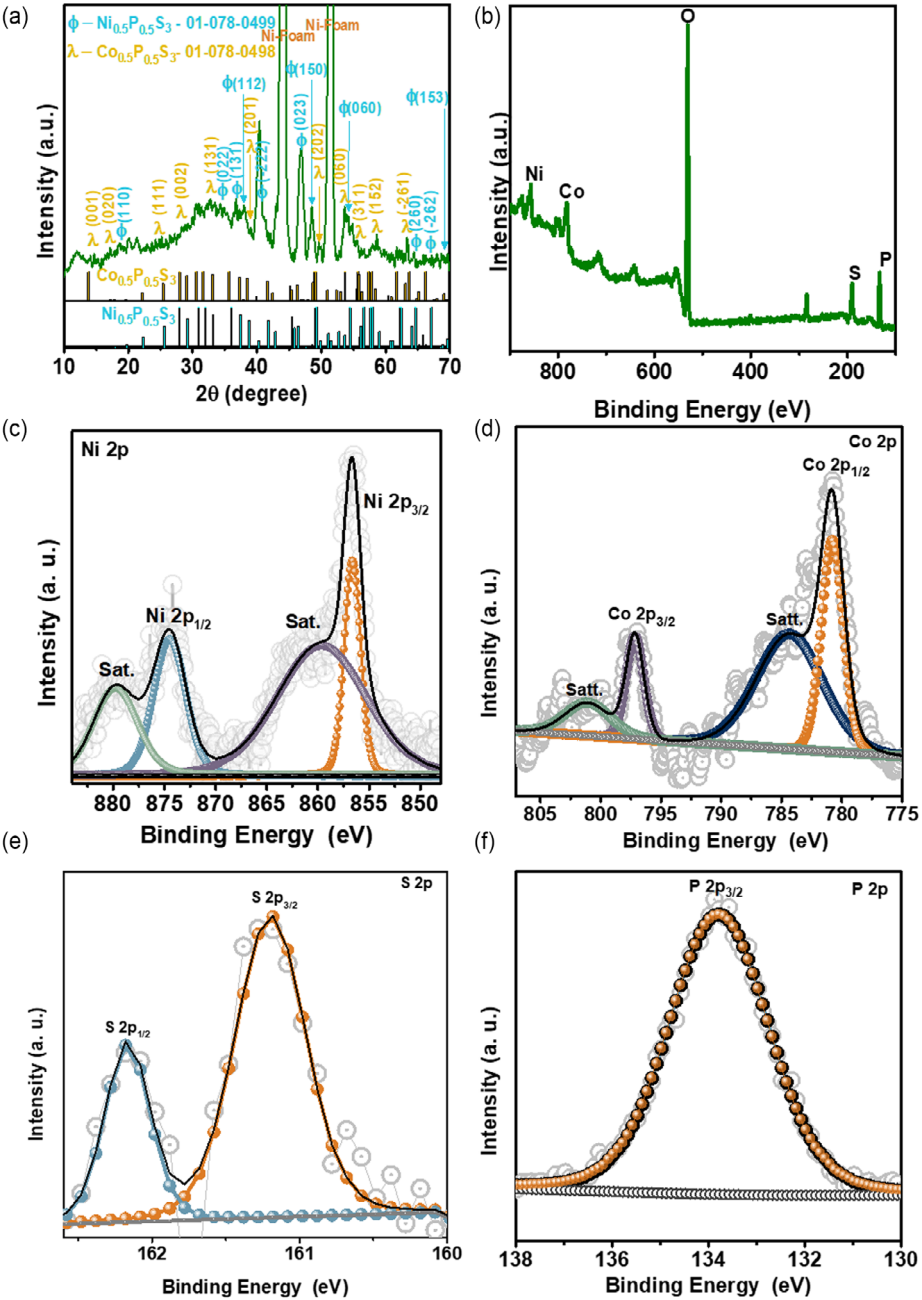
a) XRD pattern of the P@NCS_20_ material. b) Complete XPS survey scan spectrum of the P@NCS_20_ material. Core‐level XPS spectra of the c) Ni 2p, d) Co 2p, e) S 2p, and f) P 2p for the P@NCS_20_ material.

To analyze the crystallinity of the synthesized material, X‐ray diffraction (XRD) characterization was conducted. **Figure** **2**(a) shows the XRD patterns of the P@NCS_20_ material. The high‐intensity peaks of the P@NCS_20_ material revealed a multiphase composition consisting of Ni_0.5_P_0.5_S_3_ (01‐078‐0499) and Co_0.5_P_0.5_S_3_ (01‐078‐0498), in agreement with the crystallographic diffraction data set of the Joint Committee on Powder Diffraction Standards (JCPDS). Specifically, the Ni_0.5_P_0.5_S_3_ peaks, marked with a Phi symbol “*φ*”, were observed at the 2*θ* angles of 13.9° (001), 17.3° (020), 25.5° (111), 28.0° (002), 35.7° (131), 38.5° (201), 49.5° (202), 53.7° (060), 55.6° (311), 58.5° (152), and 63.5° (‐216), while the Co_0.5_P_0.5_S_3_ peaks, denoted with the lambda symbol “λ”, were detected at the 2*θ* angles of 18.2° (110), 33.3° (002), 36.1° (131), 37.5° (112), 40.5° (‐222), 48.0° (150), 54.6° (060), 64.8° (260), and 66.8° (−262) among others. The subsequent phosphorization led to discernible alterations in the crystalline phase, as evidenced by the XRD pattern of the P@NCS_20_ material. Additionally, the total XPS survey scan spectrum of the P@NCS_20_ material (Figure 2(b)) showed the presence of elements such as P, Ni, Co, and S. Furthermore, the high‐resolution XPS spectra (Figure 2(c)–(f)) exhibited distinct peaks corresponding to the oxidation states and surface chemical bonding of each element, providing insights into the composition and structure of the material. As shown in the core‐level spectrum of Ni 2p in Figure 2(c), two main peaks were observed at the binding energies of 879.6 and 856.5 eV, corresponding to the Ni 2p_1/2_ and Ni 2p_3/2_ levels, respectively.^[^ ^16^, ^17^ ^]^ In addition, two satellite peaks were observed at 879.14 and 859.9 eV, respectively. Figure 2(d) depicts the high‐resolution XPS spectrum of Co 2p, displaying two prominent peaks in addition to two satellite peaks. The primary peaks are located at binding energies of 797.0 and 780.8 eV and correspond to the Co 2p_1/2_ and Co 2p_3/2_ spin–orbit levels, respectively. These peaks confirm the presence of cobalt in mixed oxidation states, which is critical for enhancing the material's pseudocapacitive properties. The satellite peaks, which are characteristic of cobalt‐based compounds, further validate the chemical environment of the Co species.^[^ ^18^ ^]^ Two satellite peaks were observed at 801.2 and 784.5 eV. The core‐level spectrum of S 2p is shown in Figure 2(e), where two major peaks were observed at 161.1 and 162.1 eV, representing the S 2p_3/2_ and S 2p_1/2_ levels, respectively.^[^ ^19^, ^20^ ^]^ The high‐resolution P 2p XPS spectrum contained one prominent peak at 133.8 eV, which corresponds to the P 2p_3/2_ level and indicates the P—O group, as shown in Figure 2(f).^[^ ^21^, ^22^ ^]^

**Figure 3:**
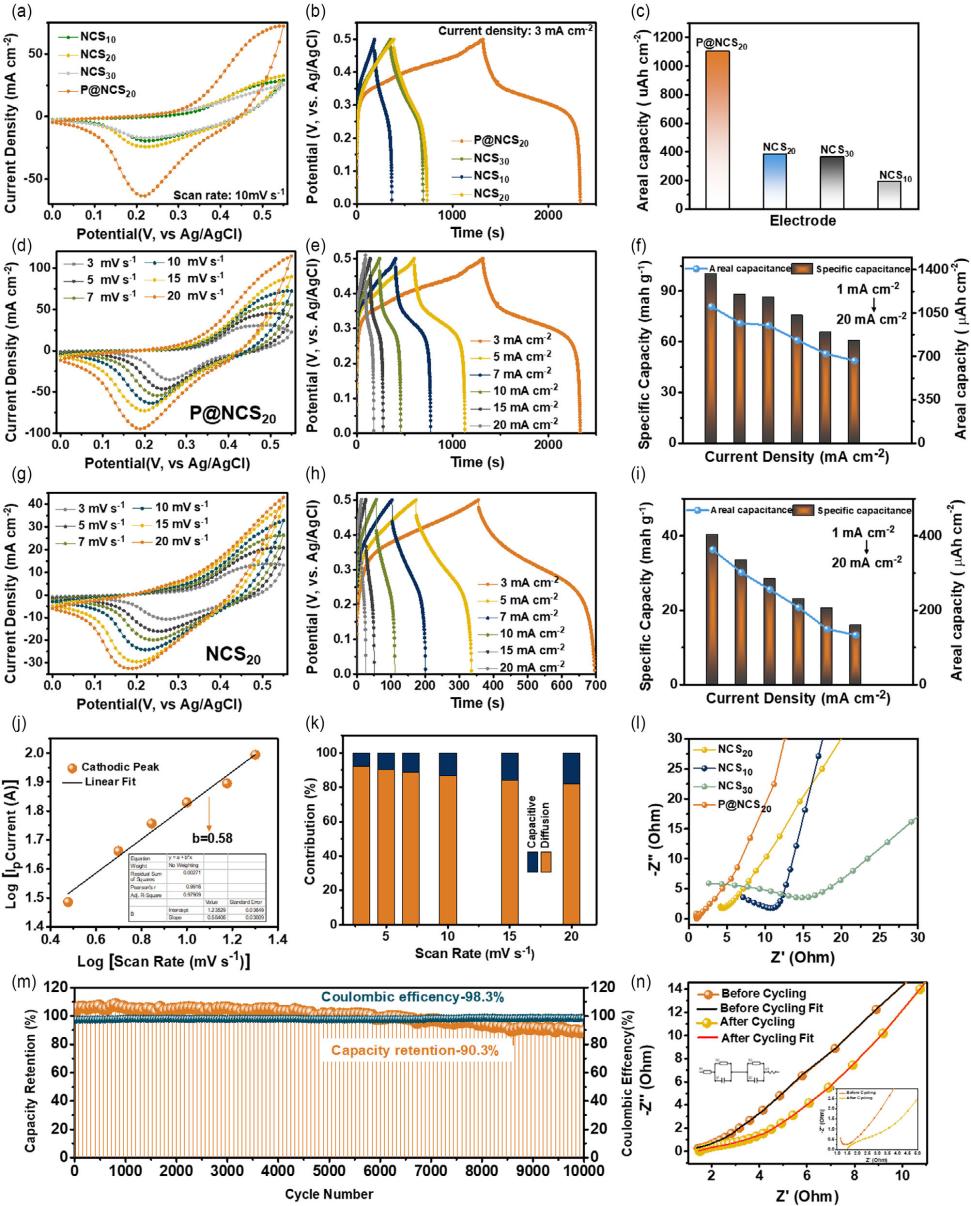
Comparative a) CV and b) GCD curves of the NCS_10_, NCS_20_, NCS_30_, and P@NCS_20_ electrodes. Comparative c) areal capacity values of NCS_10_, NCS_20_, NCS_30_, and P@NCS_20_ electrodes. d) CV curves, e) GCD curves, and f) areal capacity values of the P@NCS_20_ electrode. g) CV curves, h) GCD curves, and i) areal capacity values of the NCS_20_ electrode. j) *b* value plot for the P@NCS_20_ electrode. k) Current distribution bar graph of the P@NCS_20_ electrode. l) Comparative EIS plots of all the synthesized electrodes. m) Cycling stability test result of the P@NCS_20_ electrode for 10 000 GCD cycles. n) Fitted EIS plots of the P@NCS_20_ electrode before and after the cycling test, with the inset showing the equivalent circuit and the EIS plots at a high frequency.

**Figure** **3**(a) shows the comparative cyclic voltammetry (CV) curves of the fabricated electrodes: NCS_10_, NCS_20_, NCS_30_, and P@NCS_20_. Analysis of these curves indicates the superior oxidation and reduction behaviors of the P@NCS_20_ electrode. Compared to the other electrodes, the P@NCS_20_ electrode exhibited a larger area and a stronger redox peak intensity, indicating significantly higher activity and improved capacitance. The enhancement is directly related to the increased reactive surface area that results from metal phosphides’ lower electronegativity of P and S in the electrode matrix, which promotes redox reactions.^[^ ^23^ ^]^ The comparative GCD curves of the corresponding electrodes at a constant current density of 3 mA cm^−2^ are shown in Figure 3(b). The GCD profiles of the P@NCS_20_ electrode showed clear potential plateaus and nonlinear characteristics that match the electrochemical behavior seen in CV curves, proving the P‐coated transition metal sulfide complex.^[^ ^24^ ^]^ Concurrently, Figure 3(c) presents a bar chart illustrating the areal capacity of all the fabricated electrodes under a constant current density of 3 mA cm^−2^. As expected, the P@NCS_20_ electrode exhibited the highest areal capacity, which is attributed to its porous nanostructure and large surface area. Figure 3(d) depicts the CV curves of the P@NCS_20_ electrode, which were recorded at varying scan rates ranging from 3 to 20 mV s^−1^ within a fixed potential window of 0–0.5 V. The quasi‐rectangular shape of the CV curves, which persists across all the scan rates, indicates the predominant pseudocapacitive behavior of the electrode material. As the scan rate increases, the peak currents exhibit a proportional increase, indicating the rapid charge‐transfer kinetics and efficient ion diffusion of the electrode. This behavior confirms the material's ability to sustain high electrochemical activity, even at elevated scanning rates, which is a critical requirement for practical supercapacitor applications. The consistent electrochemical response across the tested range demonstrates the durability and suitability of the P@NCS_20_ electrode for high‐performance energy storage systems. The output current response and the peak areas gradually expanded as the scan rate increased. An increased rate of electrochemical reactions at the electrode surface may be indicated by the increasing current response with higher scan rates. Ions had enough time to interact with the electrode material's active sites during the ion diffusion process at lower scan rates. Higher current responses, however, resulted from the process switching to a more kinetically controlled regime as the scan rate increased.^[^ ^25^ ^]^ There was a noticeable increase in the redox peaks when the scan rate was increased further, from 3 to 20 mV s^−1^. This behavior suggests that the main mechanism underlying charge storage is pseudocapacitance, which is powered by Faradaic reactions. This is explained by the faster redox reactions that are taking place at the electrolyte‐active material interface. Moreover, the reduction and oxidation peaks moved to lower and higher potentials, respectively, as the scan rate increased.^[^ ^26^ ^]^ This change indicates a strong polarization effect and significant internal resistance. Figure 3(e) shows the GCD curves of the P@NCS_20_ electrode at various current densities ranging from 3 to 20 mA cm^−2^. The areal capacity values of the P@NCS_20_ electrode at various current densities, ranging from 1 to 20 mA cm^−2^, are depicted in Figure 3(f) as a combined bar and line graph. At the lowest current density of 1 mA cm^−2^, the electrode achieves an exceptional areal capacity of 1103.2 μAh cm^−2^, which gradually decreases to 665.8 μAh cm^−2^ at the highest current density of 20 mA cm^−2^. Correspondingly, the specific capacity values are 100.2 mAh g^−1^ at 1 mA cm^−2^ and 60.5 mAh g^−1^ at 20 mA cm^−2^, illustrating the material's robust performance under varying charge/discharge conditions. Figure 3(g) and (h) displays the CV and GCD curves of the hybrid electrode, respectively. The nearly unchanged shapes of these curves across different scan rates and current densities suggest fast and efficient redox reactions as well as excellent reversibility of the electrode within the tested potential window. Furthermore, Figure 3(i) presents a bar chart summarizing the areal capacities at current densities between 3 and 20 mA cm^−2^, highlighting the electrode's consistent performance. These findings confirm the P@NCS_20_ electrode's capability for rapid ion transfer and durable electrochemical stability, making it highly suitable for advanced supercapacitor applications.

The charge storage mechanism was analyzed within a defined range of b value (0.5–1), where the obtained b value of 0.58 demonstrates that the P@NCS_20_ electrode is mostly diffusion‐controlled behavior with both diffusion‐dominated and capacitive‐controlled distributions. The charge storage process in the P@NCS_20_ electrode, as shown in Figure 3(j), indicates the simultaneous presence of both diffusion‐controlled and capacitive‐controlled charge storage processes at the electrode–electrolyte interface. To further elucidate the contribution of capacitive and diffusive currents, the pseudocapacitive behavior of the P@NCS_20_ electrode was evaluated using a power law model. This model accounts for the additional capacitance arising from Faradaic reactions at the electrode surface, where the total current (i) can be expressed as^[^ ^27^, ^28^ ^]^

(1) $$i=k_{1}v+k_{2}v^{1/2}$$

This is modified to

(2) $$i/v^{1/2}=k_{1}v^{1/2}+k_{2}$$

where *k*_1_ and *k*_2_ represent the diffusive and capacitive contributions, respectively. The cathodic current contribution of the P@NCS_20_ electrode is depicted by the bar graph shown in Figure 3(k), which is calculated using Equation (6) and (7), respectively.^[^ ^27^ ^]^ The significant diffusion‐controlled contribution observed in the P@NCS_20_ electrode is attributed to the incorporation of metallic elements such as Ni and Co, alongside chalcogens (S) and phosphorus (P). The synergistic effect of these elements enhances electron and ion transports, thereby improving the charge storage kinetics due to the intrinsic electrochemical properties of the electrode material. The rapid transfer of electrons and ions significantly influences the kinetics of the reaction because of the integral and evident electrode chemistry on the surface. Notably, as the scan rate increased, the diffusion‐controlled contribution exhibited a declining trend, which positively impacted the overall electrochemical performance of the electrode. The proportion of the diffusion current contribution was calculated to be 92.19%, 90.14%, 88.55%, 86.61%, 84.08%, and 82.02% with the surface current contributions of 7.80%, 9.85%, 11.44%, 13.38%, 15.91%, and 17.93% at the scan rates of 3, 5, 7, 10, 15, and 20 mVs^−1^, respectively as shown in the bar chart of Figure 3(k). The contribution ratio of the diffusion‐controlled effect gradually decreased from 92.19% to 82.02% with the increase of scan rate. This is most likely due to the limited number of ions entering the crystal lattice. This trend indicates that at higher scan rates, charge storage becomes increasingly a mixture of both diffusion and surface currents, which is beneficial for achieving rapid charge–discharge performance in electrochemical applications.

Figure 3(l) presents the electrochemical impedance spectroscopy (EIS) plots of the synthesized NCS_10_, NCS_20_, NCS_30_, and P@NCS_20_ electrodes. The solution resistance (*R*_s_), which represents the combined resistance of the electrolyte and active material, was determined from the EIS data. Among the tested electrodes, the P@NCS_20_ electrode exhibited the lowest *R*_s_ value of 1.6 Ω, indicating superior ionic conductivity and charge transport properties. In contrast, the unmodified NCS_20_ electrode exhibited an *R*_s_ value of 4.09 Ω, while the NCS_10_ and NCS_30_ electrodes showed higher values of 7.10 and 2.59 Ω, respectively. Additionally, the charge‐transfer resistance (*R*_ct_), which reflects the resistance to electron transfer at the electrode–electrolyte interface, was also evaluated. The P@NCS_20_ electrode exhibited the lowest *R*_ct_ value of 2.03 Ω, significantly lower than those of the NCS_20_ (4.5 Ω), NCS_10_ (10.8 Ω), and NCS_30_ (15.7 Ω) electrodes. The reduced *R*_ct_ of the P@NCS_20_ electrode suggests enhanced charge‐transfer kinetics, which can be attributed to its optimized composition and structural features. This notable decrease in resistance underscores the improved electrochemical performance of the P@NCS_20_ electrode compared to its counterparts.

Cycling stability, a crucial parameter for the practical deployment of supercapacitors in electronic devices, was evaluated through continuous GCD cycles, as shown in Figure 3(m). The P@NCS_20_ electrode exhibited exceptional durability, maintaining a capacity retention of 90.3% and a Coulombic efficiency (CE) of 98.3% after 10000 cycles. This outstanding cycling performance underscores the electrode's robust structural integrity and electrochemical stability, making it a highly promising candidate for long‐term energy storage applications. EIS can provide insights into the electrochemical interface characteristics, redox kinetics, and electrode–electrolyte resistance of supercapacitors. To accurately evaluate the impedance parameters, the experimental data were fitted using a modified Randles equivalent circuit model (inset of Figure 3(n)), which consists of one (*R*_s_), charge‐transfer resistance (*R*_ct_), and two constant phase elements (CPE_1_ and CPE_2_). This model accounts for the nonideal capacitive response and distributed interfacial properties within the P@NCS_20_ architecture. The series resistance (*R*_s_), corresponding to the electrolyte's combined resistance and the electrode's internal resistance, was found to be 1.6 Ω before cycling. In comparison, the *R*_ct_ was fitted at 2.03 Ω. These low values confirm efficient ionic and electronic transport pathways in the material. After prolonged cycling, the impedance response retained a similar trend with only a modest increase in both *R*_s_ and *R*_ct_, suggesting stable electrochemical interfaces and negligible degradation. The tail in the low‐frequency region further supports a Warburg‐type diffusion process, indicating finite‐length ion diffusion into the porous matrix.

**Figure 4:**
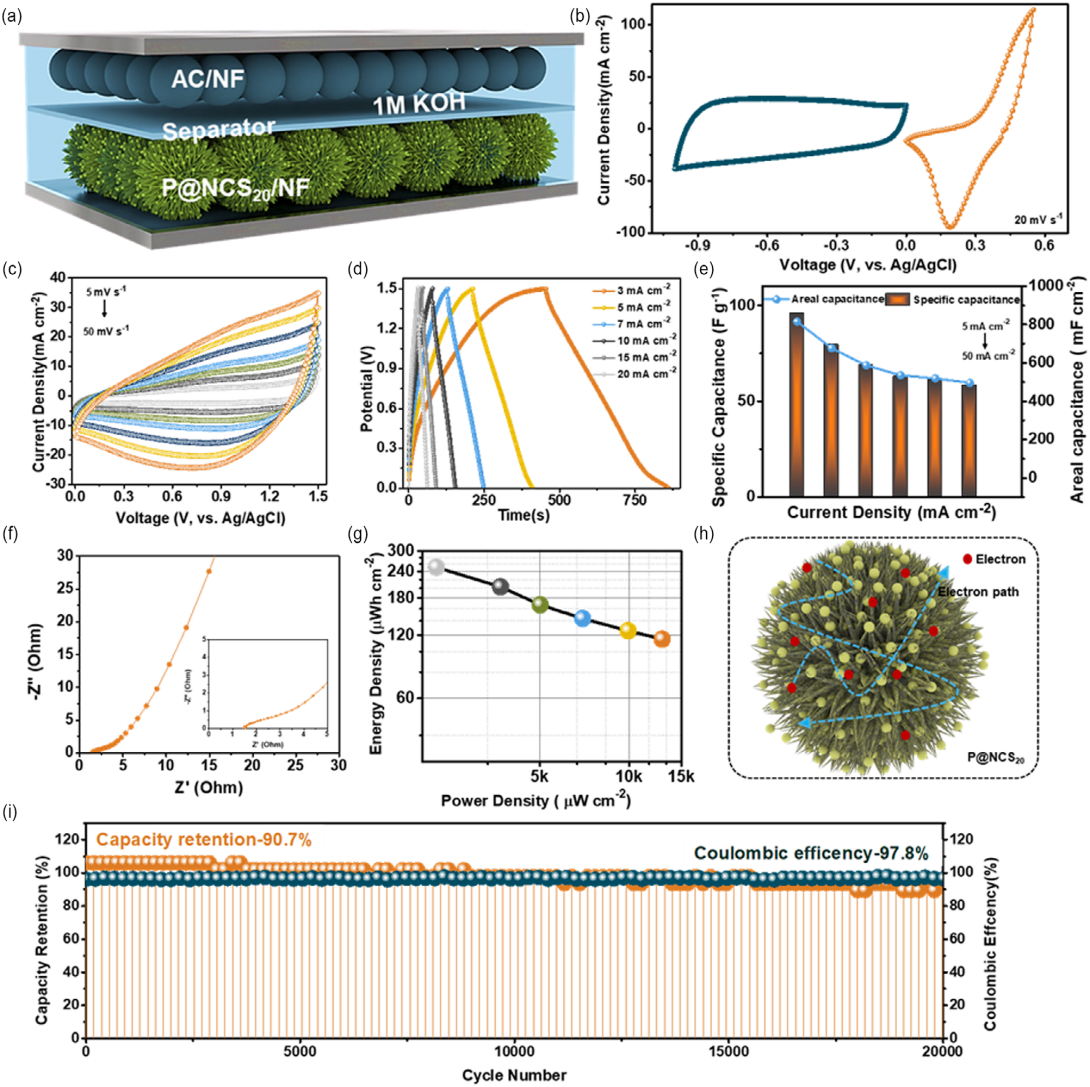
a) Schematic representation of the fabricated HSC device. b) Combined CV curves of the P@NCS_20_ and AC/NF electrodes at a scan rate of 20 mV s^−1^. c) CV curves of the HSC device at different scan rates. d) GCD curves of the HSC device at different current densities. e) Bar and line graphs for the specific and areal capacitance values of the HSC device. f) EIS plot of the HSC device. g) Ragone plot and h) schematic representation of morphological advantages of the P@NCS_20_ electrode. i) Cycling stability test result of the HSC device for 20000 GCD cycles.

The internal configuration of the HSC device is depicted in **Figure** **4**(a), emphasizing the placement of the active materials. A Whatman filter paper acts as an ion‐permeable partition between the P@NCS_20_ and AC/NF electrodes, which are positioned with their active surfaces facing one another. While avoiding direct electrical contact between the electrodes, this architecture guarantees efficient ion transport. The P@NCS_20_ and AC/NF electrodes combined with CV profiles are shown in Figure 4(b), demonstrating their complementary electrochemical behaviors. Figure 4(c) displays the CV curves of the HSC device at various scan rates, from 10 to 100 mV s^−1^, within a fixed potential window of 0–1.5 V. Even at higher scan rates, these curves maintain a nearly rectangular shape, indicating effective ion diffusion and superior pseudocapacitive performance. The GCD curves of the HSC device, which are displayed in Figure 4(d), were captured within the same potential window at current densities ranging from 3 to 20 mA cm^−2^. The device's superior charge–discharge symmetry was highlighted by the observation that the discharge times were directly proportional to the charging times across all the tested current densities. This behavior demonstrates the high performance, low internal resistance, and high electrochemical efficiency of the HSC device, which make it an excellent choice for energy storage applications requiring dependable and consistent operation.^[^ ^29^ ^]^ Across the tested current densities, the observed GCD curves indicate that the discharge time is proportional to the charging time. This behavior indicates that HSC has good charge–discharge symmetry and effective electrochemical performance.^[^ ^30^ ^]^ This behavior is indicative of an effective network for ion and electron transports within the HSC device, probably owing to its optimized composition and nanostructured morphology. According to the results displayed in Figure 4(e), the HSC device exhibited a specific (areal) capacitance of 95.93 F g^−1^ (815.4 mF cm^−2^) at an initial current density of 3 mA cm^−2^. The specific (areal) capacitance dropped to 58.31 F g^−1^ (495.65 mF cm^−2^) as the current density increased to 20 mA cm^−2^, illustrating the usual trade‐off between the capacitance and charge/discharge rate in supercapacitors. This behavior verifies the material's ability to store charge effectively at low current densities while still performing admirably at higher current densities. The EIS plot of the HSC device is shown in Figure 4(f), which indicates that the device had an *R*_s_ value of 1.86 Ω before cycling. The increased internal resistance, which is usually seen before the stabilization of electrode–electrolyte interactions during the redox processes, is the cause of this higher *R*_s_. The development of more effective electron and ion pathways at the electrode–electrolyte interface causes the internal resistance to gradually drop as the cycling continues, improving the device's overall performance. The Ragone plot of the HSC device, which displays the device's energy and power density performance, is displayed in Figure 4(g). The HSC device demonstrated an excellent balance between energy storage and rapid discharge capabilities, achieving peak values of 12900 μW cm^−2^ for areal power density and 251.4 μWh cm^−2^ for areal energy density. These findings highlight HSC's potential for high‐performance energy storage applications, where practical use requires both high energy and power densities. As seen in Figure 4(h), the schematic diagram of the P@NCS_20_ electrode's dense three‐dimensional morphological features with sharp edges and defect‐enriched surfaces greatly improves its electrochemical performance during the charge storage process.^[^ ^29^, ^31^ ^]^ Specifically, the ultraporous surface enhances charge conductivity and accommodation, shortens ion/electron transport pathways, and greatly facilitates electrolyte diffusion. These combined effects significantly enhance the overall capacity behavior and redox activity of the P@NCS_20_ electrode material.^[^ ^32^ ^]^ Additionally, the distinctive morphology of the P@NCS_20_ material facilitates effective diffusion from the top to the bottom of the nanospikes and aids in the stabilization of electrolyte ions. Therefore, the capacity performance and discharge time of the P@NCS_20_ electrode were increased due to the sea‐urchin‐like structure.^[^ ^33^ ^]^ From Figure 4(i), the HSC device had a notable retention of 90.7% after 20000 repetitions.

**Figure 5:**
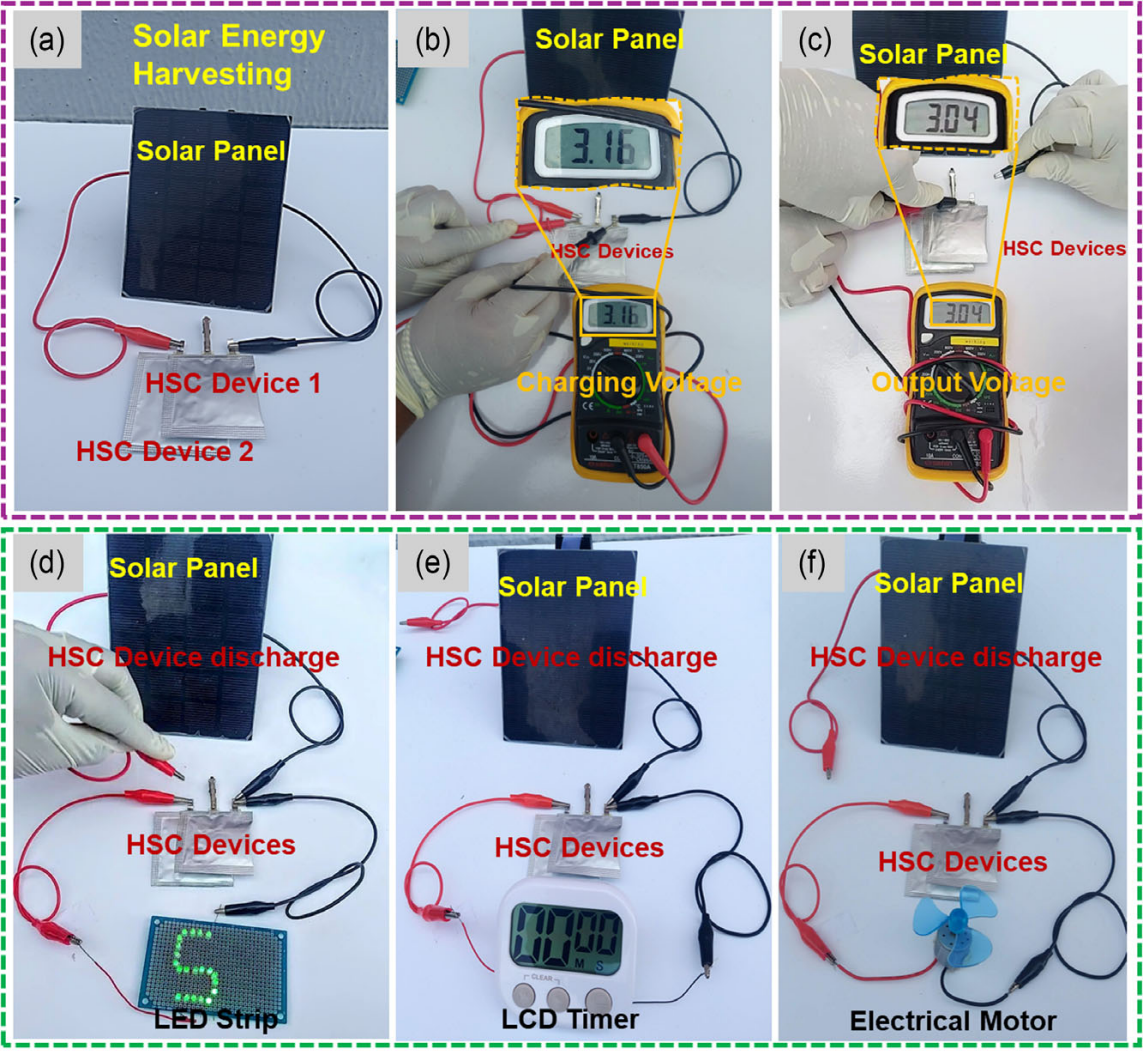
a) Photograph of two HSC devices connected in series and charged using a solar panel. b) A photograph representing the charging voltage (3.16 V) from the solar panel into the HSC devices. c) A photograph representing the output voltage from the HSC devices (3.04 V). d) Photograph of charging two series‐connected HSC devices and discharging using an LED array. Photographs of the series‐connected HSC devices powering the e) digital display timer and f) electric motor fan.

To evaluate the practical feasibility of sustainable self‐charging energy storage systems, rechargeable P@NCS_20_ and AC/NF electrodes were integrated into a renewable solar energy harvesting setup, as depicted in **Figure** **5**. Solar energy, due to its abundance and low cost, can be efficiently converted into electricity using solar panels. High‐output energy harvesting systems require efficient energy storage devices for immediate energy storage. Integrating solar energy harvesting with energy storage devices may extend the use of renewable energy even in the absence of sunlight. Supercapacitors have garnered considerable attention due to their advantageous properties. This study focuses on employing the fabricated HSC for solar energy storage to promote the integration of renewable energy sources into various applications. Figure 5(a) displays a photograph of two integrated, serially connected HSC devices assembled with a solar panel. When exposed to sunlight, the solar panel quickly charged the HSC device to an input voltage of 3.16 V, as shown in Figure 5(b). After disconnecting the solar panel, the output voltage of the HSC device was recorded at 3.04 V, as shown in Figure 5(c). Upon reaching the desired charging potential, HSC devices were integrated with light‐emitting diodes (LEDs), as illustrated in Figure 5(d). Subsequently, the charged HSC devices provided continuous power to a digital clock display and an electric motor, as depicted in Figures 5(e) and (f), demonstrating a practical application of solar charging for electronic devices. Using solar‐generated electricity, the HSC device was rapidly charged to the target voltage (3 V) within 15 s. Following the removal of the solar panel connection, the HSC devices were connected to a digital clock display and LEDs. This comprehensive harvesting‐to‐storage design illustrates the potential for developing self‐powered charging units for electronic applications.

## 3 Conclusion

Using a simple oven‐based process and phosphorus coating technique employing a tubular furnace, a novel electrode material composed of nickel (Ni), cobalt (Co), and sulfur (S) was created. Phosphorization was then added to the mixture to produce a porous nanorod structure. High areal capacity and outstanding cycle stability were among the outstanding electrochemical properties displayed by the resulting P@NCS_20_/NF electrode. During the longest discharge time, the electrode demonstrated an impressive areal capacity of 1103.2 μAh cm^−2^ in a 1 M KOH solution. After 10 000 charge/discharge cycles, the P@NCS_20_ electrode demonstrated exceptional durability with a 90.3% capacity retention and a 98.3% CE. Additionally, with the P@NCS_20_ electrode serving as the positive electrode and AC/NF as the negative electrode, the HSC device demonstrated high areal energy and power densities, with maximum values of 251.4 μWh cm^−2^ and 12900 μW cm^−2^, respectively. By effectively powering electronic devices, the device proved its usefulness and validated its suitability for practical uses. The HSC demonstrated its superior performance by achieving specific and areal capacitance values of 95.93 F g^−1^ and 815.4 mF cm^−2^, respectively, at a current density of 3 mA cm^−2^. The efficiency and sustainability of the HSC device were further showcased by its use in a variety of energy storage systems. These results suggest that the P@NCS_20_ electrodes, with their exceptional electrochemical qualities and distinctive porous sea‐urchin‐like shape, hold great promise for use in supercapacitors in the future and provide a route for the creation of more sustainable and effective energy storage technologies.

## 4 Experimental Section

4.1

4.1.1

##### Chemicals

Nickel (II) nitrate hexahydrate (Ni(NO_3_)_2_·6H_2_O), cobalt (II) nitrate hexahydrate (Co(NO_3_)_2_·6H_2_O), urea (NH_2_CONH_2_), sodium hypophosphite (NaPO_2_H_2_·H_2_O), and thioacetamide (CH_3_CSNH_2_) were procured from Sigma‐Aldrich Co. (South Korea). Additionally, N‐methyl‐2‐pyrrolidone (C_5_H_9_NO), Super P carbon black, AC powder, and polyvinylidene difluoride (‐(C_2_H_2_F_2_)_*n*_‐) were bought from Sigma‐Aldrich Co. For chemical processing, potassium hydroxide (KOH), ethanol (C_2_H_5_OH) (95%), and hydrochloric acid (HCl) were obtained from Daejung Chemicals. Laboratory‐made DIW was used as the solvent throughout the experiment.

##### Preparation of Supercapacitor Electrode Materials

To synthesize the NCS_20_ electrode materials, a solution was prepared by dissolving 0.158 g of Ni(NO_3_)_2_·6H_2_O, 0.386 g of Co(NO_3_)_2_·6H_2_O, 0.24 g of NH_2_CONH_2_, and varying concentrations (10, 20, and 30 mM) of CH_3_CSNH_2_ (sulfur (S) source) in 40 mL of DIW. The resulting solution was stirred at 100 rpm. Subsequently, a cleaned nickel (Ni) foam (NF) substrate was immersed in the growth solution, which was then transferred into an electric oven and maintained at 90 °C for 12 h. Subsequently, the NF was cleaned with ethanol and dried at 70 °C for 24 h, resulting in a powdered sample denoted as NCS_*x*_, where *x* represents the millimole (mM) concentration of the S source (i.e., CH_3_CSNH_2_) in the synthesized sample. The resulting dried NCS was then heated again to 450 °C for 2 h at a heating rate of 5 °C min^−1^. The prepared NCS_20_ electrode was then phosphorized. In this process, 0.5 g of NaH_2_PO_2_.H_2_O acted as the phosphorus source. The phosphorization was carried out in a tubular furnace under a nitrogen atmosphere at 350 °C for 3 h, with a controlled heating rate of 5 °C min^−1^. The ensuing product was designated as the P@NCS_20_ electrode.

##### Preparation of Electrode and Electrolyte

To prepare the working electrode, an NF substrate with 1 × 2 cm^2^ was cut. This substrate underwent a thorough cleaning process involving treatment with 1 M HCl, followed by acetone, and then dried in an oven at 80 °C for 30 min to ensure the removal of any residual oxide layer remnants. The electrochemical properties of the synthesized electrode materials were analyzed using CV, GCD, and EIS measurements. These analyses were conducted in a standard three‐electrode configuration using a 1 M KOH electrolyte solution with an IviumStat electrochemical workstation (IviumStat Technologies). For electrochemical analysis in a three‐electrode setup, the prepared electrodes, Ag/AgCl, and Pt wire were used as the working, reference, and counter electrodes, respectively.

##### Fabrication of HSC Device

To integrate the HSC device, a P@NCS_20_ positive electrode was paired with an AC‐coated NF (AC/NF) to serve as the negative electrode. To ensure optimal performance, mass‐balancing techniques were applied to determine the appropriate mass of AC/NF. The calculation was performed using a mass‐balancing formula. The P@NCS_20_ and AC/NF electrodes were positioned such that their active surfaces faced each other, with a Whatman filter paper acting as an ion‐permeable separator between them. This separator, characterized by numerous nanopores, facilitated the flow of ions during electrolytic activity. Ni tabs were affixed to the wires to serve as external terminals for the device. To prevent leakage, the entire setup was enclosed within a leak‐proof metal pouch filled with 1 M KOH electrolyte, and the sides were heat‐sealed for added security.

## Conflict of Interest

The authors declare no conflict of interest.

## Supporting information

Supplementary Material

## Data Availability

The data that support the findings of this study are available from the corresponding author upon reasonable request.
