# Supplementary material for: Binder‐Free Phosphorus‐Modified Multiphase Ni–Co Sulfide Nanoarchitectures with Sea‐Urchin Morphology for High‐Capacity Hybrid Supercapacitors and Practical Applications
Source: ChemSusChem. 2025 Jul 1;18(15):e202500588. doi: 10.1002/cssc.202500588 (PMC12302322; doi:10.1002/cssc.202500588)
Supplement: Supplementary file 1 — Supplementary Material [file CSSC-18-e202500588-s001.pdf]

Supporting Information

Binder-Free Phosphorus-Modified Multiphase Ni–Co Sulfide Nanoarchitectures with Sea-Urchin Morphology for High-Capacity Hybrid Supercapacitors and Practical Applications

*Ampasala Surya Kiran, Edugulla Girija Shankar, Manchi Nagaraju, and Jae Su Yu**

Department of Electronics and Information Convergence Engineering, Institute for Wearable Convergence Electronics, Kyung Hee University, 1732 Deogyeong-aero, Gihung-gu, Yongin-si, Gyeonggi-do 17104, Republic of Korea

*Address correspondence to jsyu@khu.ac.kr (J. S. Yu)

Section I: Characterization techniques

Micro-nano morphologies of materials and electrodes were observed by using a field-emission scanning electron microscope (FE-SEM; MERLIN (Carl Zeiss)) and a transmission electron microscope (TEM, JEM 200CX, JEOL). The energy-dispersive X-ray spectroscopy (EDS) was utilized to detect the elements. The crystal structure and vibrational modes of the prepared samples were investigated by X-ray diffraction (XRD, Mac Science M18XHF-SRA, Cu Kα radiation; $\lambda = 1.5406$ Å). The component and valence states of the elements were evaluated by the X-ray photoelectron spectroscopy (XPS, K-alpha (Thermo Electron)). Binding energy corrections were made to the raw spectra by using the C 1s peak at 284.6 eV.

Section II: Electrochemical measurements and necessary equations

The electrochemical properties of the electrode materials were explored using cyclic voltammetry (CV), galvanostatic charge-discharge (GCD), and electrochemical impedance spectroscopy (EIS) measurements conducted on IviumStat electrochemical workstation (Ivium Technologies) at room temperature. With a standard three-electrode configuration, 1 M KOH electrolyte and an IviumStat electrochemical workstation (IviumStat Technologies) were used to assess the electrochemical performance of each individual electrode material using CV, GCD, and EIS analyses. First, 1 M HCl and acetone were used to clean the surface of the nickel foam (NF) pieces, which had been cut into 1×2 cm^2 pieces, to prepare the working electrode. After cleaning, the NF was dried for 30 min at 80 °C in an oven to remove any remaining oxide layers. The prepared electrodes were used as the working electrode for electrochemical measurements, while platinum (Pt) wire and Ag/AgCl served as the reference and counter electrodes, respectively.

The following well-known formulae were used to determine the areal capacity values in the three-electrode configuration, as well as the specific capacitance and areal energy/power density values in the two-electrode setup:

$$C_A = \frac{2I \times \int V(t)dt}{a \times \Delta V} \quad \text{(S1)}$$

$$S_C = \frac{I \times \Delta t}{a \times \Delta V} \quad \text{(S2)}$$

$$E = \frac{I \times \int V(t)dt}{a} \quad \text{(S3)}$$

$$P = \frac{E}{\Delta t} \quad \text{(S4)}$$

where 'C_A' is the areal capacity (μAh cm^{-2}), 'I' is the applied current (A), 'Δt' is the discharge time (s), 'S_C' is the specific capacitance (F cm^{-2}), '$\int V(t)dt$' is the integral area, 'ΔV' is the potential window (V), 'E' is the areal energy density (Wh kg^{-1}), and 'P' is the areal power density (W kg^{-1}).

Section III: Morphological properties of NCS_{10} and NCS_{30} electrodes

The FE-SEM images in Figure S1 revealed the distinct surface morphologies of the NCS_{10} and NCS_{30} electrodes, which are directly correlated with their electrochemical performance. In the case of NCS_{10} (Figure S1(a)(i,ii)), the nanostructures exhibited dense needle-like features radiating outward in a well-defined spherical arrangement. While this architecture provides a relatively high surface area, the dense packing may limit electrolyte penetration and hinder rapid ion diffusion, which can compromise rate capability and result in moderate specific capacitance. Meanwhile, NCS_{30} (Figure S1(b)(i,ii)) demonstrated larger spherical clusters with more disordered and partially aggregated nanosheets. Although the increased Ni/Co precursor concentration enhances the overall growth, it leads to excessive aggregation and thicker nanosheet formation. This morphological evolution could introduce higher internal resistance and reduced electrochemically active surface area, thereby diminishing the charge storage

efficiency. The overlapping and dense stacking seen in NCS_{30} may also impede electrolyte accessibility, resulting in lower capacitance and slower charge/discharge behavior compared to the optimized sample. The observed electrochemical trends support these morphological insights: NCS_{10} shows moderate performance due to limited active site exposure, while NCS_{30}, despite the higher precursor loading, suffers from structural densification and limited ion accessibility. These results emphasize the critical role of morphology in defining the electrode–electrolyte interface kinetics and justify the superior performance observed in the NCS_{20} and $P@NCS_{20}$ electrodes, which features a more balanced nanostructure optimised for both ion transport and redox activity.

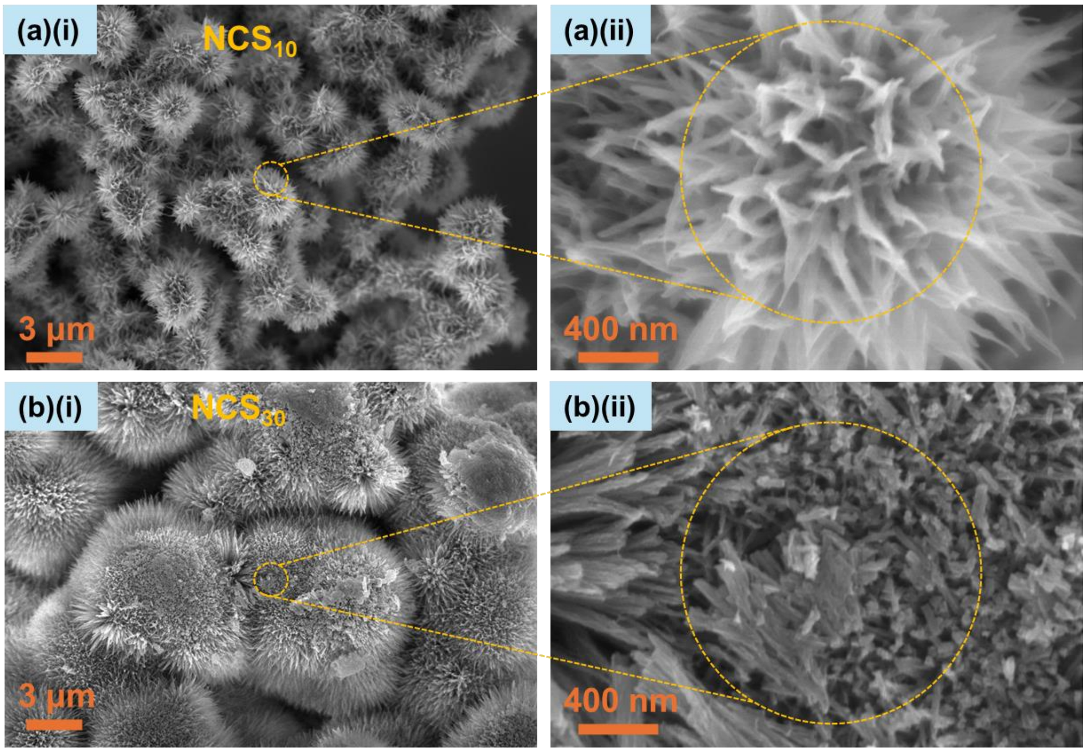

Fig. S1. (a)(i,ii) FE-SEM images of the NCS_{10} electrode. (b)(i,ii) FE-SEM images of the NCS_{30} electrode.

Table T1. Comparative capacity performances of the P@ NCS_{20} electrode with previously reported electrodes.

No.	Material	Methodology	Electrolyte	Areal capacity	Current density	Ref.
1	Co_3S_4/NF	Hydrothermal	1 M KOH	543.7 μAh cm^{-2}	1 mA cm^{-2}	1
2	O-V_2O_5@500	Pulsed laser deposition	1 M KOH	73.3 μAh cm^{-2}	2 mA cm^{-2}	2
3	NCO-NSs/CFC	Hydrothermal	3 M KOH	438 μAh cm^{-2}	1 mA cm^{-2}	3
4	NiMn-OH nanosheets	Solvothermal	1 M KOH	881 μAh cm^{-2}	3 mA cm^{-2}	4
5	NC LDH NFAs@NSs/ Ni fabric electrode	Oven-based wet chemical method	1 M KOH	536.96 μAh/cm^2	2 mA cm^{-2}	5
6	**P@NCS_{20} electrode**	**Solvothermal & Phosporization**	**1 M KOH**	**1103.2 μAh cm^{-2}**	**1 mA cm^{-2}**	**This work**

Table T2. Comparative cycling performances of the P@NCS_{20} electrode with previously reported electrodes.

No.	Material	Electrolyte	Cycling stability	No. of cycles	Ref.
1	Ni-Co-S_4	1 M KOH	90.16%	10,000	6
2	NiCoS/CC electrodes	2 M KOH	84%	3,000	7
3	$NiCo_2S_4$@MnO_x	3 M KOH	90.01%	10,000	8
4	CNTs/Ni-Co-S-3	2 M KOH	70.1%	5,000	9
5	Ni_7S_6@$NiCo_2S_4$	3 M KOH	83%	8,000	10
6	P-Co_3S_4@Ni_3S_4-175 electrode	2 M KOH	73%	3,000	11
7	P doped Co–Ni–S electrode	3 M KOH	84%	10,000	12
8	**P@NCS_{20} electrode**	**1 M KOH**	**90.3%**	**10,000**	**This work**

Section IV: Preparation of negative electrode (AC/NF)

The AC powder, PVDF, and Super P carbon black were combined in a weight ratio of 80:10:10 and meticulously ground, with a few drops of NMP added to achieve a smooth slurry consistency. This slurry was meticulously brush-coated onto the cleaned 1 × 1 cm^{-2} NF substrate and dried in an oven at 80 °C for 24 h. After drying, the electrode was pressed under 10 MPa, forming an AC/NF composite.

Section V: Electrochemical properties of AC/NF electrode

The electrochemical properties of the electrode materials were explored using CV, GCD, and EIS measurements conducted on IviumStat electrochemical workstation (Ivium Technologies) at room temperature. To evaluate the electrochemical performance of the AC/NF electrode, a three-electrode system containing an aqueous electrolyte of 1 M KOH was used. Figure S1(a) displays the AC/NF electrode's CV curves, which were obtained at scan rates between 10 and 100 mV s^{-1} within a potential window of -1 to 0 V. The CV curves' constant shapes as the scan rate rises demonstrate the electrode's exceptional electrochemical stability. At higher scan rates, there are less distortions visible in the quasi-rectangular shape of the CV curves, which indicates favorable capacitive behavior and effective ion transport. This shape, which increases in curve size as the scan rate increases, is indicative of a non-Faradaic charge storage mechanism. The GCD curves of the AC/NF electrode, recorded at current densities between 2 and 30 mA cm^{-2} in the potential range between -1 and 0 V, are shown in Figure S1(b). The curves' linearity highlights the adsorption and desorption processes that occur during cycles of charging and discharging. The AC/NF electrode demonstrated an areal capacitance of 452.72 mF cm^{-2} at an initial current density of 2 mA cm^{-2}, as shown in Figure S1(c), based on these discharge curves.

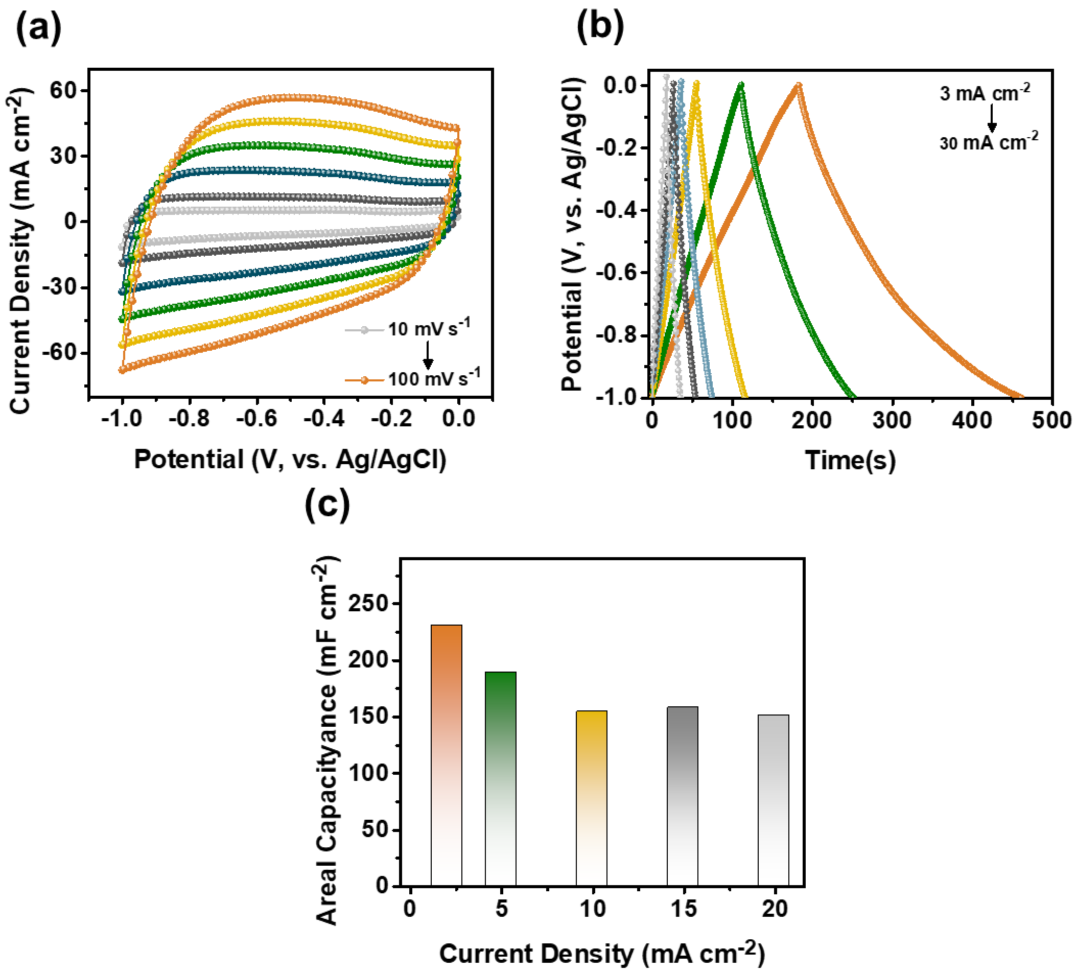

Fig. S2. Electrochemical properties of the negative electrode (AC/NF). (a) CV curves. (b) GCD curves. (c) Areal capacitance values.

Section VI: Electrochemical properties of P@NCS$_{10}$ and P@NCS$_{30}$ electrodes

The CV and GCD curves of the P@NCS$_{10}$ and P@NCS$_{30}$ electrodes are illustrated in Figures S3(a,c) and (b,d), respectively. All the electrodes exhibited quasi-rectangular CV profiles with distinct redox peaks and characteristics of pseudocapacitive behavior, indicating the contribution of Faradaic redox reactions. Among them, the P@NCS$_{20}$ electrode displayed the largest integrated CV area, suggesting a higher charge storage capability compared to the P@NCS$_{10}$ and P@NCS$_{30}$ electrodes at an identical scan rate. This enhanced performance is further evident by the GCD curves, where the P@NCS$_{20}$ electrode demonstrates the longest discharge time, reflecting superior specific capacity and excellent charge-discharge reversibility. The incorporation of P into the NCS$_{20}$ matrix plays a critical role in improving electrochemical performance. P doping introduces additional active sites and improves the electrical conductivity of the host material, thus enhancing both electron transport and ion diffusion at the electrode/electrolyte interface. Meanwhile, the P@NCS$_{10}$ electrode contained a lower P content, resulting in limited conductivity improvement, and the P@NCS$_{30}$ electrode with a higher P loading might suffer from excessive structural disruption or agglomeration, negatively affecting electrochemical kinetics. In contrast, the P@NCS$_{20}$ electrode exhibited an optimal P content, achieving a synergistic balance in conductivity, structural integrity, and redox activity. This optimized configuration facilitates faster charge transport and more efficient utilization of electroactive sites, positioning the P@NCS$_{20}$ as the most efficient and high-performing electrode among them.

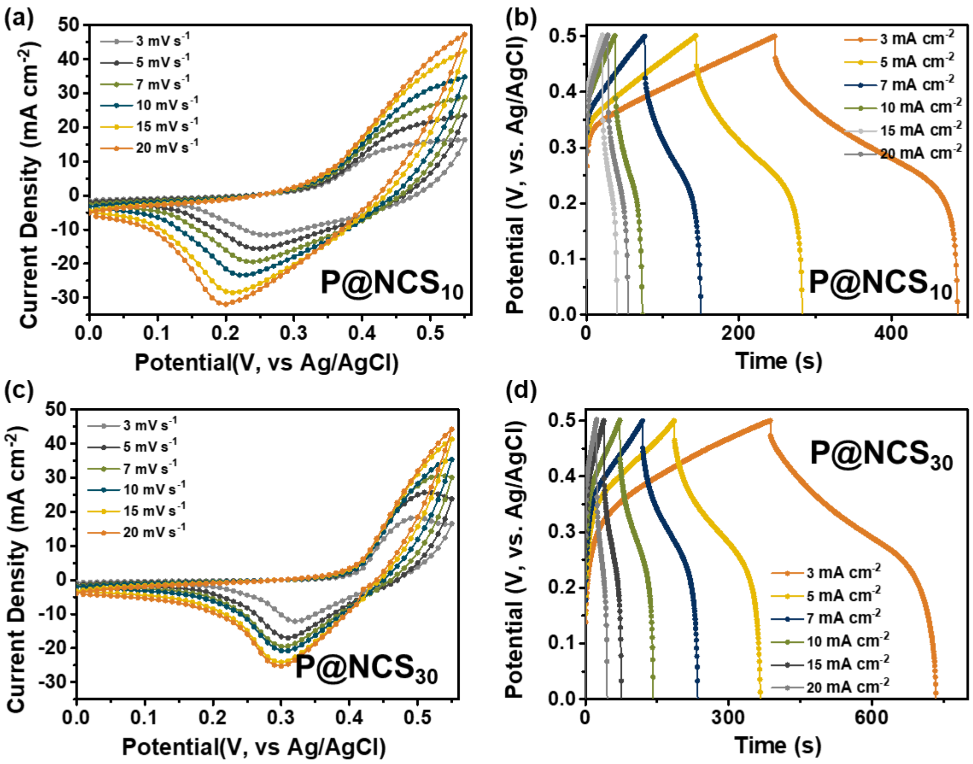

Fig. S3. (a) CV and (b) GCD curves of the P@NCS_{10} electrode. (c) CV (d) GCD curves of the P@NCS_{30} electrode.

Table T3. Comparative capacitance performance of the HSC device with previously reported devices.

No.	Material	Electrolyte	Areal capacitance	Ref.
1	AC@NF//NCS@NF	2 M KOH and 0.1 M $K_4[Fe(CN)_6]$	110 mF cm^{-2}	13
2	Ni-Co-S//rGO FSS-yarn cell	LiOH/PVA gel	181.4 mF cm^{-2}	14
3	rGO/Ni_1Co_1S	Inorganic/organic composite solid electrolyte	73.98 mF cm^{-2}	15
4	FeOOH//CFP	2 M KOH	341 mF cm^{-2}	16
5	$NiCo_2S_4$@NiCoP/NF//AC ASC	6 M KOH	429 mF/cm^2	17
**6**	**P@NCS_{20} electrode//AC HSC device**	**1 M KOH**	**815.4 mF cm^{-2}**	**This work**

Table T4. Comparative energy and power densities of the HSC device with previously reported devices.

No.	Material	Electrolyte	Energy density (E_D)	Power density (P_D)	Ref.
1	AC@NF//NCS@NF	2 M KOH and 0.1 M $K_4[Fe(CN)_6]$	33 μWh cm^{-2}	6,019 μW cm^{-2}	13
2	Ni_aCo_bS@NF//AC	2 M KOH	1.25 μWh cm^{-2}	6 mW cm^{-2}	18
3	MoS_2/PEDOT:PSS		65.8 μW cm^{-2}	0.5 mW cm^{-2}	19
4	$NiCo_2S_4$@CC// Fe_2O_3@CC	PVA-KOH gel electrolyte	93.43 μWh cm^{-2}	700.7 μW cm^{-2}	20
5	NCPS@C@G//AC ASC	3 M KOH	131.3 μWh cm^{-2}	6,400 W kg^{-1}	21
**6**	**HSC device**	**1 M KOH**	**251.4 μWh cm^{-2}**	**12,900 μW cm^{-2}**	**This work**

The atomic percentages calculated from EDS are as follows:
P: 30.95%, Co: 22.93%, Ni: 40.72% and S: 5.41%.

These results are consistent with the expected elemental distribution in the synthesized P@NCS electrode, further validating the composition and successful doping of phosphorus.

Table T5. EDS percentage of the P@NCS electrode.

Map

Element	At. No.	Mass [%]	Mass Norm. [%]	Atom [%]	abs. error [%] (3 sigma)
P	15	16.78	19.67	30.95	2.01
Co	27	23.65	27.73	22.93	2.30
Ni	28	41.84	49.05	40.72	4.04
S	16	3.03	3.56	5.41	0.42
		**85.30**	**100.00**	**100.00**	

After the cycling test, the XRD pattern continues to display distinct peaks corresponding to $Ni_{0.5}P_{0.5}S_3$ (JCPDS 01-078-0499) and $Co_{0.5}P_{0.5}S_3$ (JCPDS 01-078-0498), indicating that the crystalline phases of the active material remained intact during the cycling process. Minor broadening and intensity reduction of the peaks were observed, likely due to partial surface amorphization or stress-induced lattice disorder. Importantly, no new phases or degradation products were detected. This confirmed that the multi-crystalline framework of the active material remained largely preserved after the extended cycling. In the XPS analysis, the high-resolution spectra of Ni 2p, Co 2p, S 2p, and P 2p showed that the oxidation states and bonding environments were largely retained after cycling. In the Ni 2p spectrum (Figure 2(c)), two main peaks were observed at 879.4 and 856.7 eV binding energies, corresponding to the Ni $2p_{1/2}$ and Ni $2p_{3/2}$ levels, respectively. The core-level XPS spectrum of Co 2p displayed two prominent peaks in addition to two satellite peaks. The primary peaks were located at the binding energies of 797.1 and 780.9 eV corresponding to the Co $2p_{3/2}$ and Co $2p_{1/2}$ spin-orbit levels, respectively. The core-level XPS spectrum of S 2p showed two major peaks, observed at 161.6 and 162.7 eV, representing the S $2p_{3/2}$ and S $2p_{1/2}$ levels, respectively. The high-resolution P 2p XPS spectrum contained one prominent peak at 133.82 eV, which corresponds to the P $2p_{3/2}$. Slight shifts in binding energy, particularly in Ni and Co spectra, suggest mild surface oxidation, which is typical for pseudocapacitive materials after prolonged operation. Crucially, no significant loss of P or S signals was observed, indicating good chemical retention of the active components. These XPS results validate that no major degradation, phase transformation, or leaching of active elements occurs, thus supporting the exceptional capacity retention (90.3%) observed over 10,000 cycles. The Raman spectrum of the $P@NCS_{20}$ electrode after cycling revealed broad yet detectable bands centred around ~200 cm^{-1} and within the 550–620 cm^{-1} range. The former corresponds to metal–sulfur (M-S) vibrational modes, particularly those of Ni-S and Co-S bonds, which are characteristic of layered transition metal phosphosulfides such

as $NiPS_3$ and $CoPS_3$.[22-24] The latter region is attributed to P-S stretching vibrations and metal–phosphorus (M-P) bonding, indicating that P remains chemically integrated within the lattice structure even after prolonged electrochemical cycling. Importantly, the absence of any Raman-active modes in the 800-1000 cm^{-1} region, where metal-oxygen (M-O) vibrations such as NiO and Co_3O_4 typically appear at ~470-680 cm^{-1}, confirms that no significant oxidation or phase transformation occurs. The overall broadening and low intensity of the Raman features are the characteristics of nanostructured materials with partial crystallinity or disordered frameworks.[25] Slight peak broadening or shifting observed after cycling may reflect increased lattice disorder or partial amorphization, which is common in high-rate electrode materials. Together, these Raman features, in conjunction with consistent XRD and XPS results after the cycling test, strongly support the structural stability and chemical robustness of the $P@NCS_{20}$ electrode. This stability correlates well with its excellent electrochemical durability, including a capacity retention of 90.3% over 10,000 charge–discharge cycles. These findings are consistent with the observed electrochemical performance, where the electrode retained 90.3% of its initial capacity after 10,000 cycles. These spectroscopic studies after the cycling test support the excellent electrochemical stability and structural integrity of the $P@NCS_{20}$ electrode and validate the robustness of the Ni–Co–P–S matrix under repeated redox cycling.

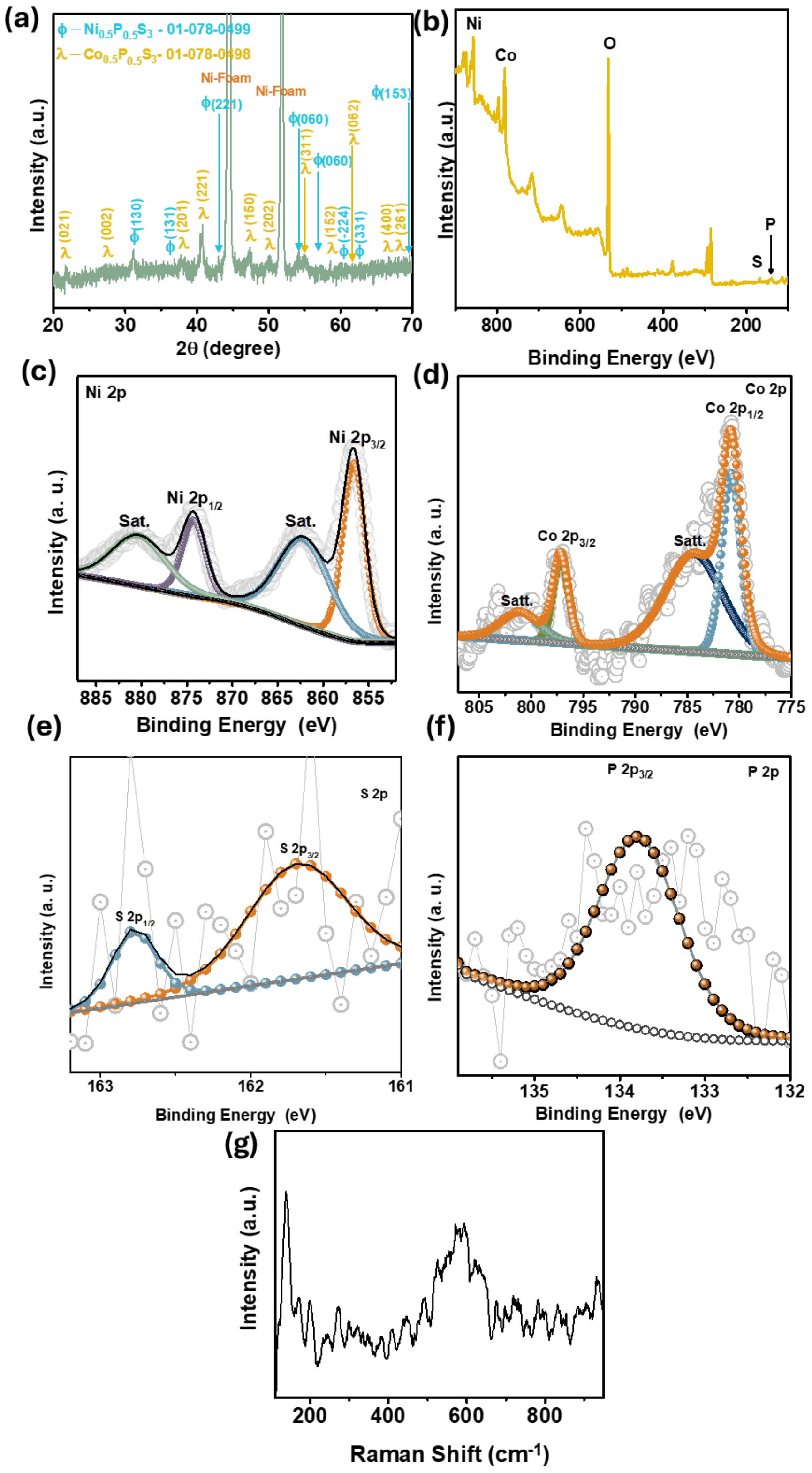

Fig. S4. (a) XRD pattern, (b-f) XPS spectra, and (g) Raman spectrum after the cycling test.

As seen in Figure S5(a)(i,ii), from the FE-SEM images before the cycling test, the P@NCS$_{20}$ electrode exhibited a well-defined and highly porous 3D nanostructure composed of interconnected nanosheets and nanorods forming sea-urchin-like assemblies. This architecture facilitates efficient ion transport and accommodates volume changes during cycling, thereby enhancing overall electrochemical performance. The FE-SEM images after the cycling test revealed that overall morphology remained largely preserved. The hierarchical sea-urchin-like structures were still distinguishable, and there was no evidence of severe structural collapse, aggregation, or pulverization. Although minor surface roughening and densification of the nanosheets could be observed at higher magnification, the electrode maintained its porous and interconnected nature, which is critical for maintaining ionic accessibility and charge storage capacity. The retention of structural features strongly correlates with the excellent cycling stability observed in the electrochemical measurements. Specifically, the P@NCS$_{20}$ electrode retained 90.3% of its initial capacity after 10,000 cycles, which can be directly attributed to the robust architecture that resists degradation under repeated redox reactions. These FE-SEM results after the cycling test provide strong visual and structural evidence supporting the mechanical and morphological stability of the electrode material, further reinforcing the reliability and practical potential of our design for high-performance supercapacitor applications, as shown in Figure S5(b)(i,ii).

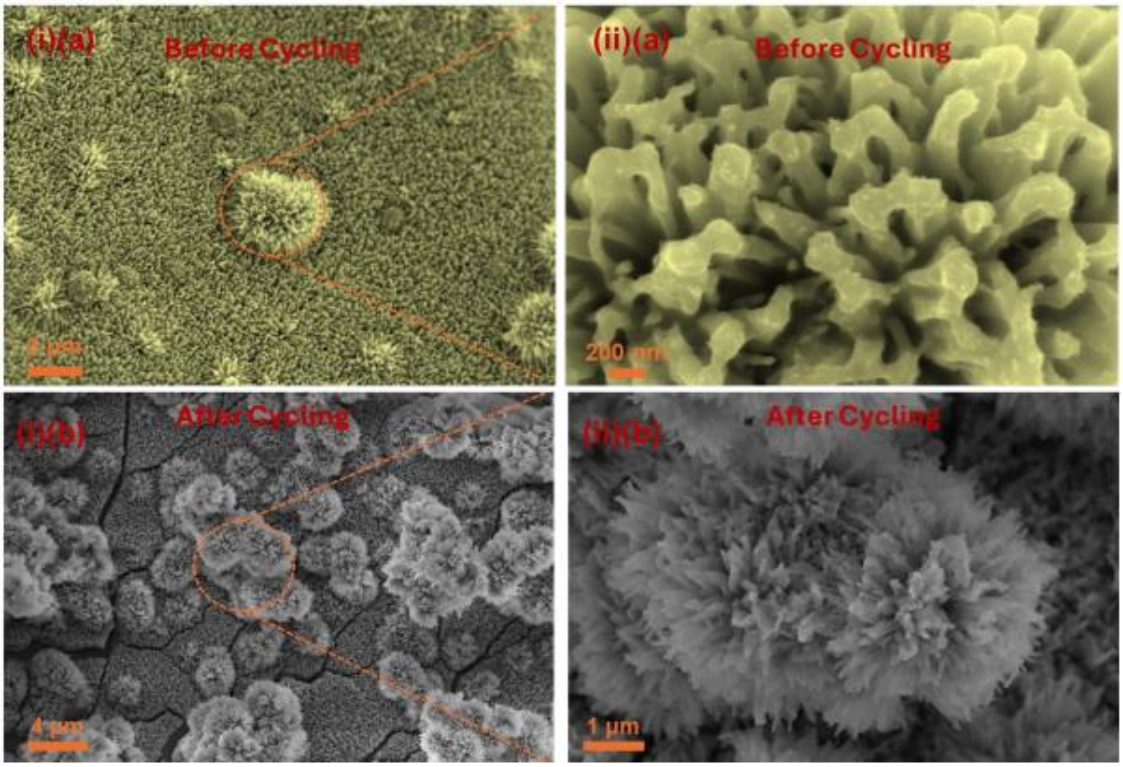

Fig. S5. FE-SEM images of the P@NCS$_{20}$ electrode (a)(i,ii) before and (b)(i,ii) after the cycling test.

References

[1] Saleem, M.S.M., Swaminathan, R., Mohan, V., Ali, N.U.H.L. and Kim, S.J., 2024. Sulfurization of cobalt oxide to cobalt sulfide: A positrode for the high-performance supercapacitor. J. Ind. Eng. Chem. 136, 493–500.

[2] Velmurugan, R., Mary, A.S., Pandikumar, A., Murugan, P. and Subramanian, B., 2024. Pulsed Laser Ablation of Oxygen deficiency Enriched Superlattice Vanadium Pentoxide (V_2O_5) Ultrathin Nextrode aiming for Flexible Binder-less Tandem Energy Harvesting Devices. Small, *20*(42), 2403531.

[3] Jiang, Y., Zhang, L., Zhang, H., Zhang, C. and Liu, S., 2016. Hierarchical $Ni_{0.54}Co_{0.46}O_2$ nanowire and nanosheet arrays grown on carbon fiber cloth for high-performance supercapacitors. J. Power Sources, *329*, 473-483.

[4] Chen, R., Xue, J., Gao, X., Yu, C., Chen, Q., Zhou, J., Sun, G. and Huang, W., 2020. Jahn–Teller distortions boost the ultrahigh areal capacity and cycling robustness of holey NiMn-hydroxide nanosheets for flexible energy storage devices. Nanoscale, *12*(43), 22075-22081.

[5] Nagaraju, G., Chandra Sekhar, S., Krishna Bharat, L. and Yu, J.S., 2017. Wearable fabrics with self-branched bimetallic layered double hydroxide coaxial nanostructures for hybrid supercapacitors. ACS Nano, *11*(11), 10860-10874.

[6] Jiang, J., Sun, Y., Chen, Y., Hu, X., Zhu, L., Chen, H. and Han, S., 2019. One-step synthesis of nickel cobalt sulfide nanostructure for high-performance supercapacitor. J. Mater. Sci., *54*, 11936-11950.

[7] Liu, T., Liu, J., Zhang, L., Cheng, B. and Yu, J., 2020. Construction of nickel cobalt sulfide nanosheet arrays on carbon cloth for performance-enhanced supercapacitor. J. Mater. Sci. Technol., *47*, 113-121.

[8] Parale, V.G., Kim, T., Patil, A.M., Phadtare, V.D., Choi, H., Dhavale, R.P., Kim, Y., Jun, S.C. and Park, H.H., 2022. Construction of hierarchical nickel cobalt sulfide@manganese oxide nanoarrays@nanosheets core-shell electrodes for high-performance electrochemical asymmetric supercapacitor. Int. J. Energy Res., *46*(4), 5250-5259.

[9] Huang, M., Zhao, K., He, D., He, J. and Wang, Y., 2022. MOF-derived nickel–cobalt sulfide nanoflakes wrapped carbon nanotubes for hybrid supercapacitors. *Energy & Fuels*, *36*(24), 15234-15243.

[10] Sui, Q., Li, J., Xiang, C., Xu, F., Zhang, J., Sun, L. and Zou, Y., 2022. Nickel metal-organic framework microspheres loaded with nickel-cobalt sulfides for supercapacitor electrode materials. J. Energy Storage, *55*, 105525.

[11] Tan, X., Feng, Z., Yang, W., Zou, H. and Chen, S., 2023. Flower-like heterogeneous phosphorus-doped Co_3S_4@Ni_3S_4 nanoparticles as a binder-free electrode for asymmetric all-solid-state supercapacitors. ACS Appl. Energy Mater., *6*(2), 702-713.
[12] Meng, Y., Sun, P., He, W., Teng, B. and Xu, X., 2019. Uniform P doped Co–Ni–S nanostructures for asymmetric supercapacitors with ultra-high energy densities. *Nanoscale*, *11*(2), 688-697.
[13] Maile, N.C., Shinde, S.K., Kim, D.Y., Devarayapalli, K.C. and Lee, D.S., 2023. Synthesis of nickel cobalt sulfide on Ni foam for improved electrochemical energy storage: Effect of binder-free reverse pulse potentiostatic electrodeposition and redox additive. J. Alloys Compd., *967*, 171845.
[14] Chen, Y., Xu, B., Wen, J., Gong, J., Hua, T., Kan, C.W. and Deng, J., 2018. Design of Novel Wearable, Stretchable, and Waterproof Cable-Type Supercapacitors Based on High-Performance Nickel Cobalt Sulfide-Coated Etching-Annealed Yarn Electrodes. Small, *14*(21), 1704373.
[15] Wang, J., Zhan, P., Zhang, D. and Tang, L., 2023. Nickel cobalt sulfide composite nanosheet anchored on rGO as effective electrode for quasi-solid supercapacitor. J. Energy Storage, *70*, 107938.
[16] Li, Y., Zhou, M., Cui, X., Yang, Y., Xiao, P., Cao, L. and Zhang, Y., 2015. Hierarchical structures of nickel, cobalt-based nanosheets and iron oxyhydroxide nanorods arrays for electrochemical capacitors. Electrochim. Acta, *161*, 137-143.
[17] Chang, X., Li, W., Liu, Y., He, M., Zheng, X., Bai, J. and Ren, Z., 2019. Hierarchical $NiCo_2S_4$@NiCoP core-shell nanocolumn arrays on nickel foam as a binder-free supercapacitor electrode with enhanced electrochemical performance. J. Colloid Interface Sci., *538*, 34-44.
[18] Chou, S.H., Lin, L.Y. and Chiu, Y.H., 2019. Pulse reverse electrodeposited nickel cobalt sulfide nanosheets on Ni foam as battery-type electrode for battery supercapacitor hybrids. J. Energy Storage, *25*, 100903.
[19] Chao, Y., Ge, Y., Chen, Z., Cui, X., Zhao, C., Wang, C. and Wallace, G.G., 2021. One-pot hydrothermal synthesis of solution-processable MoS_2/PEDOT: PSS composites for high-performance supercapacitors. ACS Appl. Mater. Interfaces, *13*(6), 7285-7296.
[20] Liang, X., He, H., Yang, X., Lü, W., Wang, L. and Li, X., 2021. In-situ growth of bimetallic sulfide $NiCo_2S_4$ nanowire on carbon cloth for asymmetric flexible supercapacitors. J. Energy Storage, *42*, 103105.

[21] Su, S., Sun, L., Qian, J., Shi, X. and Zhang, Y., 2021. Hollow bimetallic Phosphosulfide NiCo–P/S nanoparticles in a CNT/rGO framework with Interface charge redistribution for battery-type Supercapacitors. ACS Appl. Energy Mater., *5*(1), 685-696.

[22] Kuo, C.T., Neumann, M., Balamurugan, K., Park, H.J., Kang, S., Shiu, H.W., Kang, J.H., Hong, B.H., Han, M., Noh, T.W., and Park, J.G., 2016. Exfoliation and Raman Spectroscopic Fingerprint of Few-Layer $NiPS_3$ Van der Waals Crystals. Sci. Rep., *6*(1), 20904.

[23] Choi, C., Ashby, D., Rao, Y., Anber, E., Hart, J.L., Butts, D., Wilson, C., Levin, E., Taheri, M., Ghazisaeidi, M., and Dunn, B., 2022. Mechanistic Insight and Local Structure Evolution of $NiPS_3$ upon Electrochemical Lithiation. ACS Appl. Mater. Interfaces, *14*(3), 3980-3990.

[24] Rao, R., Selhorst, R., Siebenaller, R., Giordano, A.N., Conner, B.S., Rowe, E., and Susner, M.A., 2024. Mode-Selective Spin–Phonon Coupling in van der Waals Antiferromagnets. Adv. Phys. Res., *3*(6), 2300153.

[25] Bai, X., Ren, Z., Du, S., Meng, H., Wu, J., Xue, Y., Zhao, X., and Fu, H., 2017. In-situ structure reconstitution of $NiCo_2P_x$ for enhanced electrochemical water oxidation. Sci. Bull., *62*(22), 1510-1518.
